# Lifestyle interventions for depression in primary care: a qualitative study

**DOI:** 10.3399/BJGPO.2024.0233

**Published:** 2025-08-13

**Authors:** Jolien Panjer, Manna Alma, Tryntsje Fokkema, Tom Hendriks, Daniëlle Cath, Jolien Kik, Huibert Burger, Marjolein Berger

**Affiliations:** 1 Department of Primary and Long-term Care, University Medical Center Groningen, Groningen, Netherlands; 2 Department of Health Sciences, Applied Health Research, University Medical Center Groningen, University of Groningen, Groningen, Netherlands; 3 Department of Psychiatry, University Medical Center Groningen, University of Groningen, GGZ Drenthe Mental Health Services, Groningen, Netherlands; 4 GGZ Drenthe Mental Health Services Drenthe, Assen, Netherlands

**Keywords:** depression, qualitative research, health promotion

## Abstract

**Background:**

In individuals with depression a vicious cycle tends to occur in which depressive symptoms cause an unhealthy lifestyle, which reversibly causes an increase in depressive symptoms; both of which are associated with a decreased life expectancy. A potential way to break this cycle entails a multicomponent lifestyle intervention (MLI).

**Aim:**

To explore the barriers and facilitators for an MLI in patients with depressive symptoms from the perspective of GPs, chronic disease practice nurses (CD-PNs), mental health nurses (MHNs), lifestyle coaches (LC), and patients.

**Design & setting:**

Qualitative study using semi-structured interviews in Dutch primary care.

**Method:**

We interviewed five GPs, six MHNs, five CD-PNs, five LCs, and seven patients. Focus was on possible barriers and facilitators for an MLI. Data were analysed using thematic analysis. A focus group was used as a member check.

**Results:**

The following five themes were identified: expectations of effectiveness; motivation; stigma; logistics and organisation; and communication by professionals.

**Conclusion:**

Ideas on effectiveness were crucial and could be either a facilitator or a barrier for a depression-tailored MLI (DT-MLI). Professionals often had high expectations, based on work experience, making this a facilitator. Other facilitators were motivating participants, good logistics and good communication by professionals, thus destigmatising depression. Patients considered being motivated by the programme as a reason for participating, as they did not expect a DT-MLI would give them new information. Support from others was considered a motivator to participate.

## How this fits in

Lifestyle interventions are potentially effective in reducing depressive symptoms. Research on lifestyle interventions, which focuses on decreasing depressive symptoms in primary care, is rare. In this study facilitators and barriers for implementing such an intervention, among patients and primary care professionals, were identified. These can be used for further research and for motivating patients to participate in lifestyle interventions in primary care.

## Introduction

With a lifetime prevalence of 10–20% and a recurrence risk of 27.1–42%, major depressive disorder (MDD) is common in the general population.^1–3^ MDD is linked to a reduced life expectancy of nearly 10 years and lower quality of life.^4–7^ Even subclinical depression increases the risk of cardiac mortality, although to a lesser extent.^8,9^ An unhealthy lifestyle is a major contributing factor to both MDD and subclinical depression (together further referred to as depression).^10^

An unhealthy lifestyle not only often results from depression, but also feeds back into depression, creating a vicious cycle.^10–12^ Improving lifestyle, particularly through exercise, can reduce depressive symptoms, with effects comparable with antidepressants and psychotherapy.^13–15^ Even physical activity below World Health Organization (WHO)-recommended levels shows significant benefits.^16^

Most patients with depression in the Netherlands are treated in primary care by mental health nurses (MHN) and GPs, with lifestyle support also provided by chronic disease practice nurses (CD-PNs).^17,18^ If needed, they refer patients to multicomponent lifestyle interventions (MLIs) delivered by trained lifestyle coaches (LCs). MLIs have shown positive effects on physical activity, diet, and quality of life.^19–21^ Although the positive effects of lifestyle improvements on mental health are well known,^13,15,22–24^ few studies have focused on the impact of MLIs on depressive symptoms. Two meta-analyses found small effects, although many studies had high risk of bias, and the MLIs were not tailored for depression.^13,25^ Only one study in primary care evaluated depressive symptom changes from a nutrition and activity programme, with both intervention and control groups showing symptom reduction.^26^

In the Netherlands, MLIs are covered by basic health insurance, but patients with depression are often excluded owing to assumed motivational issues and barriers to participating in group interventions.^19^ Indeed, depression is linked to motivation problems.^27^ We hypothesise that MLIs can be improved with augmentations tailored to the needs of patients with depression. A qualitative study revealed that not all GPs are familiar with MLIs and some doubt their feasibility.^28^ The barriers and facilitators remain unknown for a depression-tailored MLI (DT-MLI) in primary care, which aims to reduce depressive symptoms.

Before testing a DT-MLI in primary care, we aim to reflect on barriers and facilitators by exploring the perspectives of a broad spectrum of stakeholders, including professional caregivers and patients.

## Method

### Study design

We held semi-structured interviews with GPs, MHNs, CD-PNs, LCs, and (ex)patients with depression, to assess potential benefits, barriers, and requirements of a DT-MLI. Additionally, a focus group was organised as a member check (responder validation). The study was conducted in the northern Netherlands between July 2021 and February 2022. The Consolidated Criteria for Reporting Qualitative Research (COREQ) checklist^29^ was used.

### Study context

In the Netherlands, GPs act as gatekeepers for secondary care. MHNs and CD-PNs work with the GPs and take care of patients with chronic disease and patients with mild mental healthcare problems. A GP referral is also required for insurance-covered treatment by a LC. Dutch guidelines suggest to first treat patients with mild-to-moderate depressive symptoms in primary care by giving psycho-education and brief psychological treatment by the MHN.^30^

### Participants and recruitment

Eligible for the study were GPs, MHNs, CD-PNs, LCs and (ex)patients with (a history of) depressive symptoms or disorders, aged≥18 years with sufficient ability to read and understand Dutch. We contacted GPs, MHNs, and CD-PNs from our network. LCs were identified similarly or by an internet search. Patients were identified by GPs who participated in the interviews or by other GPs from our network. Patients could have a current depressive episode or a history of depression. One practice performed a search for patients with depression in the electronicl patient files, and randomly selected 10 of these. We used snowballing sampling to identify further participants from our initial sample. For the focus group all previous participants who had consented to further interviewing were invited. Potential participants were screened for eligibility, and basic demographic data including age, educational level, years of work experience, educational background (lifestyle coaches), time of depressive episode (patients), and other demographic parameters were collected in a survey. Potential participants received an information letter and were asked to contact the researchers for planning the interview. Written informed consent was obtained beforehand, and all data were de-identified and handled anonymously.

### Data collection

Data were obtained through semi-structured interviews and a focus group. The interview guide was informed by sensitising concepts from the literature^13,25,28,31–36^ and pre-existing insights. Based on that, a topic list was developed and discussed with tailored topic lists for each stakeholder as a result containing open-ended questions on depression and lifestyle, the value and important components of a lifestyle programme, experience in treating depression, meanings attributed to lifestyle, and to the role of the participant in an MLI. Topic lists were pilot tested, with new topics added iteratively based on interviews. More details are in Supplementary List S1. Interviews were held face to face, except for three, which were held online (using Microsoft Teams) owing to COVID-19 restrictions. They entailed one-on-one conversations, except for one interview with two CD-PNs. Interviews lasted 20–67  minutes and were audio or videotaped. Field notes were added. JP (GP trainee and PhD student) conducted 25 out of 27 interviews as a researcher rather than a physician. TF (post-doc researcher) conducted one interview and TH (GP trainee and post-doc researcher) conducted another. Interviewers were trained in qualitative research and interviewing. Further characteristics of the research team can be found in Supplemental Table S2. The focus group included two CD-PNs, two MHNs, and one GP. Patients and LCs were unavailable; one patient was ill at time of the focus group, other participants did not want to participate, did not have time, or we did not get any response. Interviews were reviewed by participants for feedback and member verification in an online Microsoft Teams meeting. One researcher (JP) led the group; a second facilitator (PB, research assistant) made field notes and captured real-time comments in the chat window, inviting comments on these as appropriate. The 105-minute meeting was videotaped with consent.

### Data analysis

At a certain point, no new perspectives came up during the interviews, which was interpreted as data saturation. No official check for saturation was done. We used a content approach with thematic analysis, combining an inductive and deductive approach.^37^ Atlas.ti (version 9) was used to facilitate analysis. Verbatim interview transcriptions were made and JP coded three interviews using open coding, thereby ‘generating initial codes’. TF coded the same interviews and one additional using JP’s codebook. Codebook consensus was reached through two rounds of discussion with a third experienced researcher (MA, senior qualitative researcher, social scientist). The resulting codebook was used for coding all data (JP). Coded interviews were checked by one other researcher (MA or PB). Mind maps were created to organise and identify themes and sub-themes, then discussed with researchers and GPs. JP and MB reviewed and refined the themes by discussing mind maps, identifying overlapping codes, and arranging them into candidate and final themes. The focus group provided feedback, which was used to combine and finalise the themes. Finalised themes were discussed by JP, MB, and MA. A detailed analysis was then written. Illustrative interview citations, translated by an English native speaker, were provided.

## Results

### Participants

We interviewed seven patients, five GPs, six MHN, five CD-PN, and five LCs (Table 1). Demographic data were provided by 5/7 patients, 5/5 GPs, 5/6 MNHs, 4/5 CD-PNs, and 4/5 LCs. The onset of depression in most patients occurred 6 years ago (range 1–7  years) with its current state being unknown.

**Table 1.** Characteristics of interviewed participants

	PTs (*n* = 7)		GPs (*n* = 5)	MHN (*n* = 6)	CD-PN (*n* = 5)	LC (*n* = 5)
Female, *n/N*	5/7		2/5	4/6	5/5	4/5
Age^a^ (median [range])	54 (38–66) (*n* = 5)		51 (45–63) (*n* = 5)	55 (29–63) (*n* = 5)	47.5 (34–59) (*n* = 4)	47 (38–56) (*n* = 4)
Time of depression^a^ (years ago; median [range])	6 (1–7) years ago *(n* = 5*)*		n.a.	n.a.	n.a.	n.a.
Treatment for depression^a^ *Multiple options possible*	MHN	2/5	n.a.	n.a.	n.a.	n.a.
	Medication	1/5				
	Psychologist	4/5				
Years working as caregiver^a^ (median [range])	n.a.		13 (5–31) (*n* = 5)	34 (1.5–43) (*n* = 5)	12 (6–23) (*n* = 4)	10.5 (5–33) (*n* = 4)
Location of practice (urban or rural) *n/N*	n.a.		Urban: 4/5	Urban: 4/5	Urban: 2/4	n.a.
			Rural: 1/5	Rural: 1/5	Rural: 2/4	

^a^Data not on all participants. CD-PN = chronic disease practice nurse. LC = lifestyle coach. MHN = mental health nurse. n.a. = not applicable. PT = patient.

### Themes

**Figure 1.**
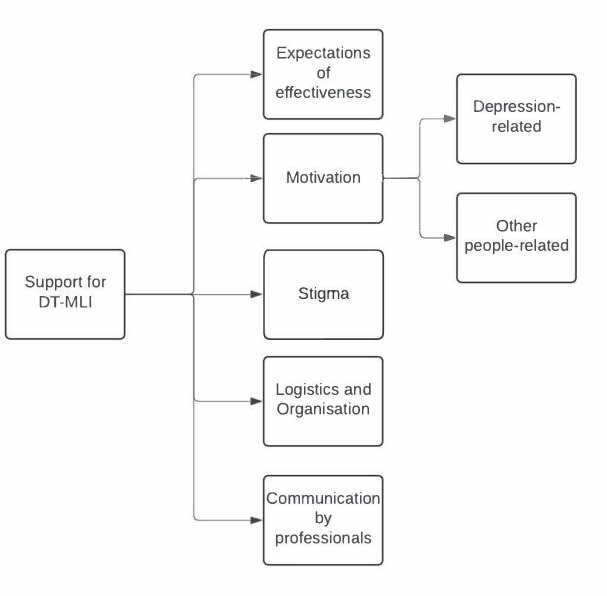
Overview of themes and sub-themes. DT-MLI = depression-tailored multicomponent lifestyle intervention

The following five main themes were identified: expectations of effectiveness; motivation; stigma; logistics and organisation; and communication by professionals with two sub-themes (Figure 1). Supplementary Table S3 shows their overview and quotes.

### Expectations of effectiveness

Ideas of the effectiveness could function as a barrier or facilitator. High expectations of effectiveness were an important facilitator and the base for support for a DT-MLI. Most GPs, CD-PNs, and MHNs interviewed had high expectations of the effectiveness of lifestyle interventions for decreasing depressive symptoms, largely based on their own clinical experience with lifestyle changes. Patients and LCs shared expectations: an MLI might provide structure in the patient’s daily lives as a possible reason for effectiveness:

*‘The basis is a basic daily schedule …. But to get a person to a point (to move) you will first have to deal with the beginning phase of such a schedule. Because if you are really depressed you won’t even get out of bed. And not even open the curtains. Let alone take a walk. So I am really convinced that that works.’* (MHN1)

One MHN mentioned that the MLI should be delivered to patients with depressive symptoms separately from other patients participating in an MLI to make them feel safer, but this could also be stigmatising. Some GPs and MHNs mentioned that a DT-MLI could create awareness of lifestyle issues in patients with depression. Some patients and MHNs thought an MLI could be a first-treatment option for depression to prevent symptom recurrence:

*‘... that depression (is) often also recurrent, that it comes back. You just offer people more if you also say "in the long run you will also get somber sometimes", and things like that can help. Then the periods when you are darker or feel gloomier get shorter and the chances of them increasing in number are also often reduced.’* (CD-PN3)

A specific DT-MLI was not necessary to some GPs, MHNs, and CD-PNs, because participants have their own specific goals within the general MLIs.

However, one GP thought that MLIs should not be offered to patients with depression. This GP believed that a general cardiovascular-focused MLI has no long-term effect (Q7, Supplementary Table 2), meaning low expectations of effectiveness could function as a barrier as well. One patient considered an MLI not effective in patients with severe depression (Q6,Supplementary Table 2):

*‘Yes, I can imagine that if you are really very depressed you will have no room for it. And that you will stop. And that would be a shame.’* (Patient [PT] 7)

Most LCs lacked experience with depressed individuals, but expected an MLI to positively impact mental health, based on experience.

Stakeholders thought that noticing progress would help patients stay in the MLI. The programme was considered helpful by enhancing motivation for lifestyle changes:

*‘I think particularly of making, a little bit for yourself, a compulsory list of daily activities, eh, that you think for yourself what you can do, but that you also think, "Oh, but now I have to". Because you soon tend to say "I don’t feel like doing anything, so I’ll just stay in bed", but then you don’t change anything.’* (PT1)

### Motivation

This theme consists of the following two sub-themes: depression-related motivation and other people-related motivation.

#### Depression-related

Professionals mentioned some inherently depression-related factors: lack of motivation, feeling tired, and lacking problem-solving skills. This could hinder patients from starting or staying in the programme and making lifestyle changes, thereby reducing its effectiveness:

*‘... First there must be a bit of motivation. And in my opinion, if a person is dead tired, then you can’t motivate them and then I can keep telling them that they have to, but then I lose touch with them.’* (MHN5)

However, they expected patients to feel motivated to stay in the programme, in particular, by exercising more. Some patients mentioned that a DT-MLI would not give them new information, but rather might be useful for other patients with depression. One patient mentioned that depressed individuals would not join the MLI to get more knowledge, but if they would, it would be to gain motivation to make lifestyle changes:

*‘I know all that. Yes, it may sound very arrogant, but I know exactly what I’m doing wrong. Really, exactly. .... then I wish I could do something about that. Yes, I would in itself and I think I would. But if you ask me, do you think that would bring you anything new, it’s maybe just that it would be like a big stick to make me participate in that or something.’* (PT6)

#### Other people-related

Some professionals and patients thought a group intervention would be more effective owing to mutual motivation. Patients mentioned that a group intervention might give them more motivation and support; for example, through buddies within the group. In contrast, GPs noted that a group intervention might scare people off. Patients mentioned that feeling unsafe within the group to talk about mental issues, would hinder them from participating:

*‘I think in particular that they (the group) motivate people a lot because they do it together, where everyone more or less has the same problem. And the big stick — the push. That it comes back every week or 2 weeks with the same group — that motivates, as I also know a lot from patients. That it does help them to tackle the problem again, that they just set goals and meet people in the group.’* (CD-PN5)

External motivators, such as a partner or someone else from the patient’s network, were considered important for motivation by both the professionals and the patients. Participants thought the CD-PN could motivate during and after the programme, by helping to maintain lifestyle changes:

*‘So, yes, my husband is really the one who drags me through a lot of deep valleys. "Come, let’s take a walk in the woods", for example. Or if the weather is nice and dry, "Shall we take a little bike ride?*" *Those little things where you say, "No, leave me alone. I want to stay at home on the couch", he is really like, "I have to get out". He’s indoors 5 days a week, so in the weekends he’s like, “I need to get out". But he is my big stick.’* (PT4)

### Stigma

Mostly professionals mentioned that an MLI specifically for patients with depression, could be stigmatising. This could be a barrier for patients to participate:

*‘Yes, because that is important. What is your goal? It mustn’t be that you think, I’m the only one here who is depressed, so then I just don’t say it. So on the one hand, you mustn’t put labels on people because then you stigmatise them. But it does make them safer, because this is a group of people who drink too much and we are all going to do something about it together; this is a group of people who are honest and say, "I am not always glad and happy in my life. We are going to try to create a few small bright spots in it”.*’ (MHN3)

### Logistics and organisation

Participants also mentioned logistical and organisational factors that could facilitate a DT-MLI.

One GP thought offering MLIs was not a task of the GP, but should be organised by municipalities, as an unhealthy lifestyle is a public health issue:

*‘But for example such an MLI, I think that’s something for the municipality. The state. I think that, that we as GPs especially … must deal with things like high blood pressure … But we can of course* [try to] *save the whole world, … but okay, you can look at this critically, especially when it’s busy … do I have to do everything? Can’t the municipality do more?’* (GP5)

However, some other GPs thought that an MLI could serve as a valuable initial treatment option for depression before referring to mental health care, in particular in view of the long waiting lists for mental health care in the Netherlands:

*‘But it would be nice for the in-between group, those that still want to do something about depressive symptoms, but don’t want to go right away to a mental health clinic. … But anyway when I consider it this way, it could be a first step.’* (MHN5)

Possible barriers mentioned by the professionals were mainly related to costs. Furthermore, barriers were a remote location, and an unfamiliar environment where the MLI would be executed. Patients did not mention any facilitators or barriers regarding organisation-related factors.

### Communication by professionals

Several facets of communication by professionals involved in the DT-MLI were considered important. Professionals mentioned a collaboration between the MHN and LC could be facilitating a DT-MLI:

*‘I think that in case of depression you should have good consultation with the person treating the depression ... this could also be the MHN. So good cooperation between healthcare professionals ... That the collaboration is going well behind the scenes. And that what everyone does and says makes sense to the person who follows the process.’* (LC5)

A possible barrier mentioned by the MHN referred to the communication skills of the coach of the MLI. The MHN noted that coaches who patronise, give their own opinion, or are judgmental are not appreciated by patients and their behaviour could be a barrier to adherence to an MLI:

*‘But it would be so nice if people say that it’s not the intention to make people feel that they aren’t doing enough, but that they know, by indicating in a professional way, that you are really offering more tools to help them feel better ... It isn’t that we don’t think you are good enough, we just want to give the person a chance.’* (MHN3)

## Discussion

### Summary

A DT-MLI has the potential to improve depressive symptoms, quality of life, and physical health. We performed this qualitative study among key stakeholders to explore barriers and facilitators for such an intervention in primary health care.

We identified the following five themes: expectations of effectiveness; motivation; stigma; logistics and organisation; and communication by professionals.

**Figure 2.**
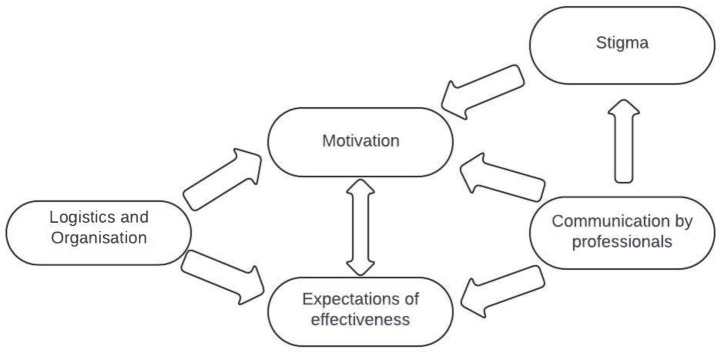
Postulated relations between themes

Most stakeholders believed that lifestyle changes positively impact mental health, highlighting the relevance of a DT-MLI in primary care. However, some professional stakeholders had low expectations, based on the limited effectiveness of cardiovascular focused MLIs. Motivation to participate in a DT-MLI can be influenced by depression-related factors such as tiredness, and other-people related factors such as group motivation. Patients considered participating in the programme mainly for motivation, not expecting new information about the importance and realisation of a healthy lifestyle. They did think it could be useful for other patients for creating awareness regarding lifestyle. Factors hampering DT-MLI implementation included stigma and logistics and organisational issues. A DT-MLI was also considered to potentially reduce waitlists for mental health care. To improve effectiveness of a DT-MLI, attention needs to be paid to the communication style of the professionals involved, ensuring it is neither judgmental nor patronising. Figure 2 depicts the postulated relationships between the themes. The expected effectiveness and participants’ motivation are reciprocally dependent, with both being influenced by logistical and organisational factors as well as the communication style of professionals. Communication is important for tackling stigma and can help increase motivation. Experienced stigma influences the motivation to participate. Optimal logistics and organisation of the DT-MLI can increase the motivation as well. These intertwined factors should be considered to increase support for a DT-MLI, and, consequently, its effectiveness.

### Strengths and limitations

A strength was that representatives of all stakeholder groups were interviewed and consequently a broad perspective was obtained. Furthermore, we used the focus group as a member check. While our stakeholders were Dutch, which may limit generalisability, many issues are likely relevant in similar countries as well. A potential limitation was the absence of patients and LCs in the focus group, which limited the member check in these groups. Our snowballing sampling could be a potential limitation, leading to mainly positive feedback if similar participants are invited to participate. Additionally, we included a selection of participants already familiar with MLIs and therefore may have been relatively positive about the potential benefits. However, we collected both positive and negative viewpoints on our topic.

### Comparison with existing literature

A recent Dutch study found that GPs recognise the value of lifestyle advice for reducing cardiovascular risk but are divided on the effectiveness of MLIs.^38^ In this and other studies, GPs expressed skepticism about MLIs’ effectiveness.^28,38^ In our study, both positive and negative views on the effect of a DT-MLI were found.

Stakeholders acknowledged the bidirectional inverse relationship between depressive symptoms and physical exercise as well as barriers to lifestyle changes in patients with mental illnesses, such as lack of motivation, stigma, and social isolation.^32,33,36^ Previous research on type 2 diabetes identified facilitators for improving lifestyle by interventions: personal goal achievement, noticeable effects, social support, and professional support,^34^ which align with our findings, although regular professional check-ups were not mentioned in our study.

Facilitators for MLIs mentioned by patients in a recent US review included noticing progress, social support, and intervention accessibility.^39^ Barriers included stigmatisation and lack of motivation. These findings corroborate our results. Because patients in this review were not selected based on depressive symptoms, these factors may be at least to some extent generic rather than depression related. Other barriers and facilitators in this review were not found in our study; for example, influence of the weather, a focus on weight and built environment (infrastructure, access to nature and so on).

Social support emerged as a common facilitator in both our study and others, showing that positive encouragement from significant others aids lifestyle changes in patients with serious mental disorders.^40^ Thus, results from our study are in line with the above mentioned studies, suggesting that most facilitators found in our study apply to MLIs in general, and are not specific for the group of depressed individuals in primary care.

### Implications for research and practice

Although our findings, combined with those from the literature, suggest that facilitators and barriers may be independent of the specific target population, complicating factors specifically related to depression may result in differences in effectiveness between individuals with and without depressive symptoms. They include lack of motivation, less expected effectiveness, and social isolation. For instance, organising social support for individuals with depression may be considerably more challenging owing to their pervasive social disturbances, which affect nearly every aspect of their social abilities.^41^ Moreover, the barrier of reduced motivation and negative outcome expectations specifically concerns depressed individuals.^42^ Thus, these factors should be taken into account in designing MLIs for depressed individuals; for example, by focusing more on finding social support or motivational techniques of professionals.^14^ Additionally, delivering the MLI in a separate group with fellow patients with depression, might help dealing with the stigma surrounding depression.

Importantly, patients mentioned a DT-MLI could be useful for others, but not for themselves, creating a form of ‘othering’. This concept can be problematic when implementing a DT-MLI. Not much is known yet about this ‘othering’ in this target population. It could be that patients have an incorrect perception of their own lifestyle issues or do not see any need for changing their lifestyle. A possible solution is to focus specifically on the motivational effect of a DT-MLI, alongside gaining knowledge on lifestyle. When communicating with patients, it might be important to focus not on why or how they should change their lifestyle (which can feel patronising), but on helping them achieve personal lifestyle goals (which is more motivating). Support from a buddy and specific psycho-education on depression may also be needed to inspire motivation for starting with and staying part of an MLI. Our study highlights the need for sensitive communication around mental illness owing to stigma, and the importance of community or personal support networks.

A barrier to implementing a DT-MLI in primary care is professionals’ skepticism towards the effectiveness of regular MLIs, which are focused on cardiovascular risk reduction.^19,43,44^ Although reduction in depressive symptoms shows more promise,^13,15,22–24,26^ doubts about effectiveness may be generalised to DT-MLIs, decreasing support and motivation (see Figure 2). Therefore, it is crucial to evaluate the ideal form and effectiveness of a DT-MLI in primary care, and to familiarise professionals with the concept as a treatment for depression before wide-scale implementation.

Our findings can inform the design and implementation of DT-MLIs. A DT-MLI with added motivational elements could provide an additional treatment option in primary care. With long waiting lists in the mental health sector in the Netherlands and other countries,^45^ a DT-MLI could help alleviate the mental healthcare crisis in primary and specialised care. Owing to the explorative nature of our study, we could have missed some barriers and facilitators. Further research is needed to give more insight on support for a DT-MLI in primary care.

To conclude, our qualitative study found several barriers and facilitators for an MLI for depression in Dutch primary care, giving an insight in what is needed to create support for a DT-MLI.

## Data

The dataset relied on in this article is available from the corresponding author on reasonable request.

## References

[1] Gerber M, Beck J, Brand S (2019). The impact of lifestyle Physical Activity Counselling in IN-PATients with major depressive disorders on physical activity, cardiorespiratory fitness, depression, and cardiovascular health risk markers: study protocol for a randomized controlled trial. Trials.

[2] Hardeveld F, Spijker J, De Graaf R (2013). Recurrence of major depressive disorder and its predictors in the general population: results from the Netherlands Mental Health Survey and Incidence Study (NEMESIS). Psychol Med.

[3] Ten Have M, de Graaf R, van Dorsselaer S (2018). Recurrence and chronicity of major depressive disorder and their risk indicators in a population cohort. Acta Psychiatr Scand.

[4] Laursen TM, Munk-Olsen T, Nordentoft M, Mortensen PB (2007). Increased mortality among patients admitted with major psychiatric disorders: a register-based study comparing mortality in unipolar depressive disorder, bipolar affective disorder, schizoaffective disorder, and schizophrenia. J Clin Psychiatry.

[5] Cuijpers P, Smit F (2002). Excess mortality in depression: a meta-analysis of community studies. J Affect Disord.

[6] Laursen TM, Musliner KL, Benros ME (2016). Mortality and life expectancy in persons with severe unipolar depression. J Affect Disord.

[7] De Graaf R, Bijl RV, Ravelli A (2002). Predictors of first incidence of DSM-III-R psychiatric disorders in the general population: findings from the Netherlands Mental Health Survey and Incidence Study. Acta Psychiatr Scand.

[8] Quadackers DMC, Cath DC, Liemburg EJ (2021). Anxiety and mood disorders are independent risk factors for cardiovascular diseases. Ned Tijdschr Geneeskd.

[9] Penninx BW, Beekman AT, Honig A (2001). Depression and cardiac mortality: results from a community-based longitudinal study. Arch Gen Psychiatry.

[10] Sarris J, O’Neil A, Coulson CE (2014). Lifestyle medicine for depression. BMC Psychiatry.

[11] Wang X, Arafa A, Liu K (2021). Combined healthy lifestyle and depressive symptoms: a meta-analysis of observational studies. J Affect Disord.

[12] Schuch F, Vancampfort D, Firth J (2017). Physical activity and sedentary behavior in people with major depressive disorder: a systematic review and meta-analysis. J Affect Disord.

[13] Wong V-H, Ho F-Y, Shi N-K (2021). Lifestyle medicine for depression: a meta-analysis of randomized controlled trials. J Affect Disord.

[14] Knapen J, Vancampfort D, Moriën Y, Marchal Y (2015). Exercise therapy improves both mental and physical health in patients with major depression. Disabil Rehabil.

[15] Verhoeven JE, Han LKM, Lever-van Milligen BA (2023). Antidepressants or running therapy: comparing effects on mental and physical health in patients with depression and anxiety disorders. J Affect Disord.

[16] Pearce M, Garcia L, Abbas A (2022). Association between physical activity and risk of depression: a systematic review and meta-analysis. JAMA Psychiatry.

[17] Cuijpers P, Quero S, Dowrick C, Arroll B (2019). Psychological treatment of depression in primary care: recent developments. Curr Psychiatry Rep.

[18] Magnée T, de Beurs DP, Boxem R (2017). Potential for substitution of mental health care towards family practices: an observational study. BMC Fam Pract.

[19] van Rinsum CE, Gerards SMPL, Rutten GM (2018). The coaching on lifestyle (CooL) intervention for obesity, a study protocol for an action-oriented mixed-methods study. BMC Public Health.

[20] Hassan Y, Head V, Jacob D (2016). Lifestyle interventions for weight loss in adults with severe obesity: a systematic review. Clin Obes.

[21] Gillies CL, Abrams KR, Lambert PC (2007). Pharmacological and lifestyle interventions to prevent or delay type 2 diabetes in people with impaired glucose tolerance: systematic review and meta-analysis. BMJ.

[22] Goracci A, Rucci P, Forgione RN (2016). Development, acceptability and efficacy of a standardized healthy lifestyle intervention in recurrent depression. J Affect Disord.

[23] Jacka FN, O’Neil A, Opie R (2017). A randomised controlled trial of dietary improvement for adults with major depression (the “SMILES” trial). BMC Med.

[24] Schuch FB, Vancampfort D, Rosenbaum S (2016). Exercise for depression in older adults: a meta-analysis of randomized controlled trials adjusting for publication bias. Braz J Psychiatry.

[25] Gómez-Gómez I, Bellón JÁ, Resurrección DM (2020). Effectiveness of universal multiple-risk lifestyle interventions in reducing depressive symptoms: systematic review and meta-analysis. Prev Med.

[26] Forsyth A, Deane FP, Williams P (2015). A lifestyle intervention for primary care patients with depression and anxiety: a randomised controlled trial. Psychiatry Res.

[27] Callaghan CK, Rouine J, O’Mara SM (2018). Potential roles for opioid receptors in motivation and major depressive disorder. Prog Brain Res.

[28] Scheenhart N, Azdahic A, Metting E (2021). Prevention: a task for the general practitioner? (In Dutch). Huisarts Wet.

[29] Tong A, Sainsbury P, Craig J (2007). Consolidated criteria for reporting qualitative research (COREQ): a 32-item checklist for interviews and focus groups. Int J Qual Health Care.

[30] Claassen N, Groeneweg BF, Heineman H (2019). [NHG standard: Depression] NHG-standaard: Depressie (in Dutch).

[31] van Rinsum C, Gerards S, Rutten G (2018). Coaching on Lifestyle (CooL) intervention: the lifestyle coach as linchpin? (in Dutch). Tijdschr Gezondheidswet.

[32] Manger S (2019). Lifestyle interventions for mental health. Aust J Gen Pract.

[33] Bauer IE, Kiropoulos LA, Crist NP (2018). A qualitative study investigating bipolar patients’ expectations of a lifestyle intervention: a self-management program. Arch Psychiatr Nurs.

[34] Schmidt SK, Hemmestad L, MacDonald CS (2020). Motivation and barriers to maintaining lifestyle changes in patients with type 2 diabetes after an intensive lifestyle intervention (the U-TURN trial): a longitudinal qualitative study. Int J Environ Res Public Health.

[35] Bos V, Dale D, Leenaars KEF (2019). Effective components of combined lifestyle interventions (in Dutch).

[36] Bouma AJ (2018). The barrier-belief approach: a new perspective of changing behavior in primary care [Thesis fully internal (DIV)].

[37] van der Heiden W, Lacroix J, Moll van Charante EP, Beune E (2022). GPs’ views on the implementation of combined lifestyle interventions in primary care in the Netherlands: a qualitative study. BMJ Open.

[38] Deslippe AL, Soanes A, Bouchaud CC (2023). Barriers and facilitators to diet, physical activity and lifestyle behavior intervention adherence: a qualitative systematic review of the literature. Int J Behav Nutr Phys Act.

[39] Aschbrenner KA, Mueser KT, Bartels SJ, Pratt SI (2013). Perceived social support for diet and exercise among persons with serious mental illness enrolled in a healthy lifestyle intervention. Psychiatr Rehabil J.

[40] Kupferberg A, Bicks L, Hasler G (2016). Social functioning in major depressive disorder. Neurosci Biobehav Rev.

[41] Krämer LV, Helmes AW, Seelig H (2014). Correlates of reduced exercise behaviour in depression: the role of motivational and volitional deficits. Psychol Health.

[42] Berendsen BA, Hendriks MR, Verhagen EA (2011). Effectiveness and cost-effectiveness of “BeweegKuur”, a combined lifestyle intervention in the Netherlands: rationale, design and methods of a randomized controlled trial. BMC Public Health.

[43] Duijzer G, Haveman-Nies A, Jansen SC (2017). Effect and maintenance of the SLIMMER diabetes prevention lifestyle intervention in Dutch primary healthcare: a randomised controlled trial. Nutr Diabetes.

[44] Punton G, Dodd AL, McNeill A (2022). “You’re on the waiting list”: an interpretive phenomenological analysis of young adults’ experiences of waiting lists within mental health services in the UK. PLoS One.

[45] Nederlandse Zorgautoristeit (2024). Mental health care waiting times remain high: people are waiting too long in almost all regions and for all diagnoses (in Dutch).

